# Language profile of children from a quilombola community

**DOI:** 10.1590/2317-1782/20212020048

**Published:** 2021-12-20

**Authors:** Talita Trigueiro Domingos, Antonélia Barros de Carvalho, Ana Manhani Cáceres-Assenço

**Affiliations:** 1 Centro de Educação e Pesquisa em Saúde Anita Garibaldi, Instituto Santos Dumont – ISD - Macaíba (RN), Brasil.; 2 Laboratório de Desenvolvimento da Linguagem, Departamento de Fonoaudiologia, Centro de Ciências da Saúde, Universidade Federal do Rio Grande do Norte – UFRN - Natal (RN), Brasil.

**Keywords:** Language Development, Language, Child Development, Language Tests, Socioeconomic Factors

## Abstract

**Purpose:**

to identify linguistic performance (expressive vocabulary, phonology and narrative) and cognitive performance (Verbal Short-Term Memory (VSTM)) of preschoolers living in a quilombola community.

**Methods:**

Twenty-four quilombola preschoolers aged four (4) and five (5) years with no complaints in language development participated in the study. Most families were in the D-E class and maternal and paternal education was lower than high school. Their guardians answered a questionnaire about their previous development, family practices and socioeconomic aspects, while the assessment included tests of expressive vocabulary, phonology, narrative and verbal short-term memory. The data collected were subjected to descriptive statistical analysis to characterize family practices, socioeconomic aspects and linguistic and cognitive performance, inferential analysis used Fisher's exact test to compare performance between subjects aged 4 and 5 years and also to compare performance according to family practices.

**Results:**

78.3% of preschoolers performed adequately in vocabulary and 79.2% in phonology; and 63.6% had the narrative classified as descriptive. 82.6% had a VSTM task performance below the expected for age.

**Conclusion:**

Although the preschoolers in this study had functional communication, their profile of language development and cognitive skills was more vulnerable and may have an impact on their school trajectory.

## INTRODUCTION

Language development is pointed out as an indicator of child well-being, as it contributes to the ability to manage emotions, communicate feelings, establish and maintain relationships, think symbolically and learn to read and write^(1)^.

Socioeconomic aspects and family cultural diversity influence the child's development^(2,3)^. A study on child development and its determinants showed below-average cognitive and linguistic performance among children whose families come from socioeconomically vulnerable areas^(4)^.

The economic level and the family structure, the educational levels of the parents and the ethnic group are causally interrelated in the sense that they affect each other, influencing the full development of the child^(5)^. A stimulating family environment is associated with a greater expressive vocabulary in childhood^(6,7)^, and the increase the level of education of parents reduces the chance of their children presenting impairments in language development^(8)^. The school environment also indicates that the performance of children in vocabulary, Short-Term Memory (STM) and narrative skills tasks is influenced by the socioeconomic level, with benefits for those who attend private institutions^(9-11)^.

In addition to the socioeconomic issue, another important issue to understand is how language development occurs in individuals coming from different cultures, as linguistic diversity can accompany cultural diversity^(12)^. However, studies that seek to investigate this relationship are scarce, which can result in difficulties in the linguistic assessment process of the child^(13)^.

The quilombolas are one of the traditional populations from Brazil and consist of an ethnic and minority group within the Brazilian black population, often residing in rural areas far from large urban centers. Due to the intense social inequalities to which it is exposed and the geographic location of the communities, this population still suffers from difficulty in accessing quality health and educational services^(14)^, which can negatively interfere with language development and increase social vulnerability.

Seeking to distinguish cultural linguistic variations from language impairments that directly affect the development of the child enables both the detection of possible situations of language disorders, as well as actions and intervention programs in childhood^(15)^.

Thus, considering the absence of studies focusing on linguistic development in the context of quilombola communities, this study aimed at identifying the linguistic (expressive vocabulary, phonology and narrative) and cognitive (Verbal Short-Term Memory (VSTM)) performance of preschoolers residing in a quilombola community, taking into account their age and family practices.

## METHODS

The study was carried out at Capoeiras a quilombola community located in Macaíba – in the state of Rio Grande do Norte (RN), where around 326 families currently live, totaling approximately 1500 people. The community has an association, a cooperative, catholic and evangelical churches, two flour producing houses, a school that serves as kindergarten and elementary school, a health center and a cultural point, which is a space where people honor the contributions of Afro culture and religious rituals.

The ethical aspects of the study were approved by the Research Ethics Committee of the Onofre Lopes University Hospital (*Comitê de Ética em Pesquisa – Hospital Universitário Onofre Lopes* (CEP - HUOL)), Federal University of Rio Grande do Norte (*Universidade Federal do Rio Grande do Norte* (UFRN)), number CAAE 07867019.2.0000.5292. The participation of the children was authorized by their parents and/or guardians by signing the Informed Consent Form (ICF) – *Termo de Consentimento Livre e Esclarecido* (TCLE).

### Participants

Data collection took place between May and December 2019 and 24 preschool children aged between four (4) years (45.8%) and five (5) years (54.2%) participated in this research, whose average was 58.6 (±6.4) months. In the 4-year-old group, 58% were female, while in the 5-year-old group, only 25% were female. All attended the same public educational institution in the community and there was no family complaint regarding language development.

As for socioeconomic data, families varied between classes C2 and D-E, being mostly D-E (86.4%). Maternal education varied between incomplete elementary school and higher education, with a predominance of incomplete elementary school and complete elementary school (30.4% each); while paternal education varied between illiterate and complete high school, with a predominance of complete elementary school (47.1%). In all families, the most frequent profession of mothers and fathers was related to agriculture (37.5%).

### Materials and procedures

Initially, an authorization was obtained from the City Hall and from the leaders of the quilombola community. Afterwards, the community health agents invited the families to hold meetings with the parents of the selected preschoolers in order to clarify the study procedures and also to assure them about the anonymity and confidentiality of the information obtained.

After obtaining consent, the parents answered a short questionnaire about the development of the child and practices in the family environment, as well as questions related to purchasing power, access to goods and services and education, therefore allowing to define their socioeconomic classification as per the Brazilian Association of Research Companies (*Associação Brasileira de Empresas de Pesquisa* (ABEP))^(16)^.

To be able to trace the children the language profile, a battery assessment was carried out, which included the video recording of expressive vocabulary, phonology, narrative and verbal short-term memory tasks. Data collection took place individually and was carried out in the community, with an average duration of 40 minutes, always following the same applied order.

To assess the vocabulary, the ABFW^(17)^ Expressive Vocabulary test was used and to comply with the objectives of this study, only responses classified as Usual Verbal Designation (UDV) were considered, both in each of the semantic fields and in total in the test.

To assess the phonological system, the naming test of the ABFW^(17)^ phonology test was used. To characterize such performance, the focus on productive phonological processes was chosen, so it was considered adequate when there was no maintenance of any productive phonological process beyond the expected overcoming age, nor occurrence of those considered unexpected in the development. On the other hand, the performance was considered altered when there was maintenance of a productive phonological process beyond the expected overcoming age and/or occurrence of processes considered unexpected in the development.

To assess VSTM, the non-word repetition test was used, consisting of 40 invented words, equally divided into mono, di, tri and polysyllabic^(18)^. As proposed by the authors, for each repetition corresponding to the target word or that differed only by productive phonological exchanges (previously analyzed according to the phonological assessment) 1 point was assigned. Next, the correct answers in each part of the test (monosyllables, disyllables, trisyllables and polysyllables) and in the total of the test (maximum of 40) were added. The percentages of correct answers, both partial and total, were calculated and the performance was classified as adequate or below expectations according to the values proposed by the test.

To elicit the narrative, the book “Frog, where are you?” ^(19)^ was used, which is composed of 25 scenes in black and white, without written words and arranged in a logical sequence. The child was asked to explore the pages and then narrate a story based on the book in hand. The speech samples were classified according to the type of narrative, namely: descriptive, causal or intentional^(20)^.

For all tests, the performance was transcribed separately by two different researchers and when a divergence was found between the analyses, a third researcher examined it, thus ensuring the accuracy of the data obtained.

### Data analysis

The data obtained were tabulated and analyzed using the SPSS version 21 statistical software. Categorical variables (socioeconomic level, education, practices in the family environment, performance in each assessment, type of narrative) were described by frequency of occurrence and percentage. Numerical variables (vocabulary, phonology, verbal short-term memory) were subjected to verification of data normality and, as they did not respect the normal distribution, they were described using the median and interquartile range.

The preschool children performance data, age groups and family practices were compared using Fisher's exact test with Bonferroni correction for multiple comparisons. For these analyses, it was necessary to reclassify the data on discourse and family practices, ensuring that we would have only two categories to compare. In the case of discourse, causal and intentional classifications were grouped; and in the case of family practices, the answers “Sometimes and never” and “Often and always” were grouped. The significance level adopted was 5%.

## RESULTS

### Descriptive analysis

Table 1 shows data related to family practices, with emphasis to the fact that 52.6% of guardians say they sometimes read to the child and 70.0% say that the child never reads books alone. Table 2 presents data related to the items that the child has and it is possible to verify that none of them have access to a computer at home (Table 2).

**Table 1:** Frequency distribution of the occurrence of family practices

	Never (%)	Sometimes (%)	Often (%)	Always (%)
Help with homework	11.1	44.4	5.6	38.9
Check homework	0.0	27.8	5.6	66.7
Read to the child	10.5	52.6	10.5	26.3
Child reads	70.0	15.0	5.0	10.0

**Table 2:** Frequency distribution of items that the preschool has access

Items	%
Musical Instruments	33.3
Board games	26.3
Dictionary	14.3
Books	47.6
Computer	0.0
Games	47.6

Regarding general performance in different language areas, the majority showed adequate performance in vocabulary (78.3%), in phonology (79.2%), and narrative classified as descriptive (63.6%), followed by causal (31.8%). As for VSTM, the majority performed below expectations for their age (82.6%).

### Comparison between ages and family practices

When comparing the performance classification in each area between the age groups, only phonology and VSTM differed, with the 5-year-old group having greater impairment (Table 3).

**Tabela 3:** Comparison of preschool performance ratings in each language assessment

Variables	Classifications	Four (4) years of age (n,%)		Five (5) years of age (n,%)		*p*
Vocabulary	Adequate	8	72.7	11	84.6	0.415
	Below expected	3	27.3	2	15.4	
Phonology	Adequate	5	45.5	0	0.0	0.011*
	Impaired	6	54.5	13	100.0	
Verbal Short-Term Memory (VSTM)	Adequate	4	40.0	0	0.0	0.024*
	Below expected	6	60.0	13	100.0	
Narrative	Descriptive	7	70.0	7	58.3	0.454
	Causal or intentional	3	30.0	5	41.7	

* Statistical difference (*p*=0.05) – Fisher's exact test

To compare the performance rating in each area between family practices related to schoolwork and reading, the Fisher's exact test with a Bonferroni correction was used for ten planned effects, resulting in a significance level of 0.005. None of these comparisons were statistically different (Tables 4 and 5).

**Table 4:** Comparison the preschooler performance classification in each language assessment according to the classification of family practices related to schoolwork.

Variável	Classification	Help with homework				p	Check homework				p
		Sometimes or never (n,%)		Often or always (n,%)			Sometimes or never (n,%)		Often or always (n,%)		
Vocabulary	Adequate	8	80.0	7	87.5	0.588	5	100.0	10	76.9	0.350
	Below expected	2	20.0	1	12.5		0	0.0	3	23.1	
Phonology	Adequate	1	10.0	2	25	0.412	1	20.0	2	15.4	0.650
	Impaired	9	90.0	6	75		4	80.0	11	84.6	
Verbal Short-Term Memory (VSTM)	Adequate	3	30.0	1	12.5	0.382	3	60.0	1	7.7	0.044
	Below expected	7	70.0	7	87.5		2	40.0	12	92.3	
Narrative	Descriptive	5	54.6	5	71.4	0.451	2	40.0	8	72.7	0.242
	Causal or intentional	4	44.4	2	28.6		3	60.0	3	27.3	

* Statistical difference with Bonferroni correction (*p* < 0.005) – Fisher's exact test

**Table 5:** Comparison of the classification of preschoolers performance in each language assessment according to the classification of family practices related to reading.

Variables	Classifications	Read to the child					*p*	Child reads			p	
		Sometimes or never (n,%)	Often or always (n,%)					Sometimes or never (n,%)	Oftenor always (n,%)			
Vocabulary	Adequate	10		83.3	6	84.2	0.704	12	70.6	3	100.0	0.399	
	Below expected	2		16.7	1	15.8		5	29.4	0	0.0		
Phonology	Adequate	3		25.0	1	14.3	0.525	3	17.6	1	33.3	0.509	
	Impaired	9		75.0	6	85.7		14	82.4	2	66.7		
Verbal Short-Term Memory (VSTM)	Adequate	4		33.3	0	0.0	0.128	4	23.5	0	0	0.491	
	Below expected	8		66.7	7	100.0		13	76.5	3	100		
Narrative	Descriptive	7		63.6	4	66.7	0.661	9	56.3	3	100	0.227	
	Causal or intentional	4		36.4	2	33.3		7	43.8	0	0		

* Statistical difference with Bonferroni correction (*p* <0.005) - Fisher's exact test

## DISCUSSION

This study aimed at tracing the linguistic performance profile of quilombola preschoolers in tests of expressive vocabulary, phonology, narrative and VSTM.

First of all, it is important to emphasize that we are dealing with a population considered to be low-income, mostly from class D-E, and with low family education, a minority of mothers and fathers who have completed high school. This information alone alerts us to possible consequences on language development, as variables such as vocabulary are identified as sensitive to the environment^(6)^.

When considering performance in language assessment, most preschoolers showed adequate performance for their age group in the expressive vocabulary test, according to the reference parameters. This result seems to contradict the literature, however there is still no evidence that the test used is sensitive and adequate to the local reality, which could mask minor damages. It is also noteworthy that about 20% of preschoolers performed below expectations and the comparison between ages, although not different, indicated a lower adequate percentage in the 4-year-old group. Although the quality of the school environment has not been considered, it was possible that their inclusion in the school has influenced in the expansion of their vocabulary^(6,21)^.

In phonology, over 75% preschoolers showed the maintenance of productive phonological processes beyond the expected overcoming age and were considered to have altered performance. When comparing the ages, there was a statistical difference, indicating that at five (5) years of age, all had altered performance, that is, they had processes that should have already been overcome. These data indicate that most children in this sample have a performance characteristic of a phonological disorder, although phonological processes related to the sonority trait have not been observed.

Even though it is not a purely linguistic measure, one of the objectives of the study was to investigate VSTM, as it is directly related to language acquisition, understanding and learning^(22,23)^. Most preschoolers performed below expectations on the non-words repetition task, and no 5-year-old subject was classified as adequate. Here, it is important to emphasize that the task used proposes to disregard the productive phonological processes as errors^(18)^, which implies that this damage cannot be explained by phonology, but is related to the storage and retrieval of phonological information.

This finding needs to be interpreted with caution, as, on the one hand, the assessment was based only on one task, on the other hand, there was a wide range of international literature pointing out that damages of this nature can impact the course of language development and school life^(24)^and are considered even as a clinical mark of developmental language disorder^(25)^. Regardless of the nature of this impairment and associated with phonological performance, this group is at risk of facing difficulties in the literacy and schooling process, as phonological processing skills play a crucial role in learning at the beginning of formal education^(26)^.

Regarding the narrative, most preschoolers produced a descriptive, a pattern that was maintained when considering the age groups. Although we do not have Brazilian reference data, the descriptive discourse is simple and does not demonstrate refinement of narrative skills^(20)^, which could already be present in the age group studied.

Here, it is interesting to point out a qualitative aspect, as a book with pictures was used to elicit the narrative, and the lack of familiarity of the participants with the handling of a book caught our attention. When interviewed about family practices, 52.2% of mothers stated that they “sometimes” read to the child and 46.7% stated that the child has books, while 70.0% stated that the child never reads books alone. It is important to emphasize that an effective reading is not expected at these ages, but that the child would be able to handle the book properly. Although this information is based on reports, the lack of familiarity with books seems to be associated with the low level of parents education and, consequently, with the value that is attributed to such practices in this community.

The absence of statistical differences between the frequency of positive family practices and linguistic performance in this sample seems to be related both to the restricted number of subjects and the need to regroup the data, as well as the fact that families present certain homogeneity with regard to socioeconomic variables. The quantity and quality of stimuli that the child receives depend, however, on their living conditions and the characteristics of the community in which they live^(27)^. Considering that it is possible to observe differences in the learning process in children who grow up in families that encourage contact with the written language^(28)^, this is another finding that reinforces the vulnerability related to the development of communicative skills of preschoolers in this community.

Overall, these findings point to the importance of investments both in monitoring communicative development in early childhood and in implementing intervention programs at the ideal age that can minimize the impact of a less favorable environment on the development of children in this community. Programs based on response to intervention in partnership with education, for example, seem to be a low-cost alternative that could bring benefits to the community^(29)^.

An analysis that was not possible at this time, but which seems promising to us, is the comparison with the performance of a group residing in the capital of the state where the study was carried out and which presents greater socioeconomic variability. This analysis will help us to understand if the instruments used may have interfered too much in these results, after all, it is important to consider the effectiveness of linguistic performance tests for population minorities due to major regional and cultural variability^(30)^.

As this is a cross-sectional study carried out with only one assessment of each child, it was not possible to further investigate the cultural influence and language development. However, the results shown here make it possible to indicate that preschoolers living in this quilombola community have a linguistic development that allows them to have a functional communication, despite there being indications of risk for difficulties in the school insertion process. Thus, considering that this is a vulnerable socioeconomic context, we highlight the importance of investing in monitoring and stimulation programs, as well as relying on the support from the school.

Finally, this study could bring a potential contribution to clinical practice in regions with less government investment and highlight the importance of thinking about public policies for early childhood aimed at developing the full potential of children in populations at risk for cognitive impairments and language disorders.

## CONCLUSION

The preschoolers in this study showed performance compatible with the reference values in vocabulary, but their phonological system showed impairments due to the maintenance of productive phonological processes, their narrative skills were descriptive and their performance in phonological STM was impaired. Even though these children have functional communication, they have a more vulnerable language and cognitive skills development profile that can affect their school trajectory.

## References

[1] Law J, Charlton J, Asmussen K (2017). Language as a child wellbeing.

[2] Piccolo LR, Arteche AX, Fonseca RP, Grassi-Oliveira R, Salles JF (2016). Influence of family socioeconomic status on IQ, language, memory and executive functions of Brazilian children. Psicol Reflex Crit.

[3] Southwood F (2013). Towards a dialect-neutral assessment instrument for the language skills of Afrikaans-speaking children: the role of socioeconomic status. J Child Lang.

[4] Neves KR, Morais RLS, Teixeira RA, Pinto PAF (2016). Growth and development and their environmental and biological determinants. J Pediatr (Rio J).

[5] Huston AC, Bentley AC (2010). Human development in societal context. Annu Rev Psychol.

[6] Moretti TCF, Kuroishi RCS, Mandrá PP (2017). Vocabulary of preschool children with typical language development and socioeducational variables. CoDAS.

[7] Hart BB, Risley TR (2003). The early catastrophe. Am Educ.

[8] Athayde ML, Mota HB, Mezzomo CL (2010). Vocabulário expressivo de crianças com desenvolvimento fonológico normal e desviante. Pro Fono.

[9] Sá CBA, Maioli MCBP, Ramos TS, Melo DFP, Macedo EC, Mecca TP (2018). Avaliação de memória de curto prazo em crianças no início do ensino fundamental. Cad Pós-Grad Distúrb Desenvolv..

[10] Brancalioni AR, Zauza A, Karlinski CD, Quitaiski LF, Thomaz MFO (2018). Desempenho do vocabulário expressivo de pré-escolares de 4 a 5 anos da rede pública e particular de ensino. Audiol Commun Res.

[11] Gardner-Neblett N, Iruka IU (2015). Oral narrative skills: explaining the language-emergent literacy link by race/ethnicity and SES. Dev Psychol.

[12] ASHA: American Speech and Hearing Association (2014). A Cultural competence.

[13] McNeilly LG (2019). Strategies utilized by speech-language pathologists to effectively address the communication needs of migrant school-age children. Folia Phoniatr Logop..

[14] Freitas DA, Caballero AD, Marques AS, Hernández CIV, Antunes SLNO (2011). Saúde e comunidades quilombolas: uma revisão da literatura. Rev CEFAC.

[15] Scopel RR, Souza VC, Lemos SMA (2011). A influência do ambiente familiar e escolar na aquisição e no desenvolvimento da linguagem: revisão de literatura. Rev CEFAC.

[16] ABEP: Associação Brasileira de Empresas de Pesquisa (2016). Critério Brasil 2015 e atualização da distribuição de classes para 2016.

[17] Andrade CRF, Befi-Lopes DM, Wertzner HF, Fernandes FDM (2004). ABFW: teste de linguagem infantil nas áreas de fonologia, vocabulário, fluência e pragmática.

[18] Rodrigues A, Befi-Lopes DM (2013). Short-term phonological memory in preschool children. CoDAS.

[19] Mayer M (1996). Frog, where are you?..

[20] Bento ACP, Befi-Lopes DM (2010). Organização e narração de histórias por escolares em desenvolvimento típico de linguagem. Pro Fono.

[21] Gilkerson J, Richards JA, Warren SF, Montgomery JK, Greenwood CR, Oller DK (2017). Mapping the early language environment using all-day recordings and automated analysis. Am J Speech Lang Pathol.

[22] Abreu PMJE, Abreu N, Nikaedo CC, Puglisi ML, Tourinho CJ, Miranda MC (2014). Executive functioning and reading achievement in school: a study of Brazilian children assessed by their teachers as “poor readers”. Front Psychol.

[23] Baddeley AD, Hitch G (1974). Working memory. Psychol Learn Motiv.

[24] Alloway TP, Gathercole SE, Kirkwood H, Elliott J (2009). The cognitive and behavioral characteristics of children with low working memory. Child Dev.

[25] Newbury DF, Bishop DVM, Monaco AP (2005). Genetic influences on language impairment and phonological short-term memory. Trends Cogn Sci..

[26] Alloway TP, Alloway RG, Wootan S (2014). Home sweet home: does where you live matter to working memory and other cognitive skills?. J Exp Child Psychol.

[27] Puglisi ML, Hulme C, Hamilton LG, Snowling MJ (2017). The home literacy environment is a correlate, but perhaps not a cause, of variations in children’s language and literacy development. Sci Stud Read.

[28] Leite KKA, Bittencourt ZZLC, Silva IR (2015). Fatores socioculturais envolvidos no processo de aquisição da linguagem escrita. Rev CEFAC.

[29] Miranda MC, Piza CT, Assenço AMC, Villachan-Lyra P, Pires IAH, Silva ECC (2019). Adaptação do Modelo Pre-K RTI ao contexto brasileiro da educação infantil: desafios e perspectivas. Neuropsicol Latinoam..

[30] Medeiros VP, Valença RKL, Guimarães JATL, Costa RCC (2013). Vocabulário expressivo e variáveis regionais em uma amostra de escolares de Maceió. Audiol..

