# Supplementary material for: Language profile of children from a quilombola community
Source: Codas. 2021 Dec 20;34(2):e20200048. doi: 10.1590/2317-1782/20212020048 (PMC9769429; doi:10.1590/2317-1782/20212020048)
Supplement: Translation

# Translation: Perfil de linguagem de pré-escolares de uma comunidade quilombola

**Authors:** Talita Trigueiro Domingos, Antonélia Barros de Carvalho, Ana Manhani Cáceres-Assenço

**Affiliations:** 1 Centro de Educação e Pesquisa em Saúde Anita Garibaldi, Instituto Santos Dumont – ISD - Macaíba (RN), Brasil.; 2 Laboratório de Desenvolvimento da Linguagem, Departamento de Fonoaudiologia, Centro de Ciências da Saúde, Universidade Federal do Rio Grande do Norte – UFRN - Natal (RN), Brasil.

**Keywords:** Desenvolvimento da Linguagem, Linguagem, Desenvolvimento Infantil, Testes de Linguagem, Fatores Socioeconômicos

## Abstract

**Objetivo:**

identificar o desempenho linguístico (vocabulário expressivo, fonologia e discurso) e cognitivo (memória de curto prazo verbal) de pré-escolares residentes de uma comunidade quilombola.

**Método:**

Vinte e quatro pré-escolares quilombolas de 4 e 5 anos sem queixas no desenvolvimento da linguagem participaram do estudo. A maioria das famílias era da classe D-E e a escolaridade materna e paterna era inferior ao Ensino Médio. Seus responsáveis responderam a um questionário sobre seu desenvolvimento prévio, práticas familiares e aspectos socioeconômicos, enquanto a avaliação incluiu testes de vocabulário expressivo, fonologia, discurso e memória de curto prazo verbal. Os dados coletados foram submetidos à análise estatística descritiva para caracterizar as práticas familiares, os aspectos socioeconômicos e o desempenho linguístico e cognitivo. A análise inferencial utilizou o teste exato de Fisher para comparar o desempenho entre os sujeitos de 4 e 5 anos, além de comparar o desempenho de acordo com as práticas familiares.

**Resultados:**

Maior parte dos pré-escolares apresentou desempenho adequado no vocabulário (78,3%) e na fonologia (79,2%); e 63,6% tiveram o discurso classificado como descritivo. 82,6% apresentaram desempenho em tarefa de memória de curto prazo verbal abaixo do esperado para idade.

**Conclusão:**

Ainda que os pré-escolares deste estudo apresentem comunicação funcional, seu perfil de desenvolvimento da linguagem e de habilidades cognitivas demonstrou ser mais vulnerável, o que poderá repercutir em sua trajetória escolar.

## INTRODUÇÃO

O desenvolvimento da linguagem é apontado como um indicador de bem estar infantil, pois contribui para a capacidade de gerenciar emoções, comunicar sentimentos, estabelecer e manter relacionamentos, pensar simbolicamente e aprender a ler e a escrever^(1)^.

Aspectos socioeconômicos e diversidade cultural familiar influenciam no desenvolvimento da criança^(2,3)^. Um estudo sobre o desenvolvimento infantil e seus determinantes mostrou desempenho cognitivo e linguístico abaixo da média entre crianças cujas famílias eram de áreas socioeconomicamente vulneráveis^(4)^.

O nível econômico e a estrutura familiar, os níveis educacionais dos pais e o grupo étnico estão inter-relacionados causalmente no sentido de que afetam um ao outro, influenciando o pleno desenvolvimento criança^(5)^. Um ambiente familiar estimulador está associado a um maior vocabulário expressivo na infância^(6,7)^, e o aumento do grau de escolaridade dos pais reduz a chance dos filhos apresentarem alterações no desenvolvimento da linguagem^(8)^. O ambiente escolar também indica que o desempenho de crianças em tarefas de vocabulário, memória de curto prazo e habilidades narrativas sofre influências do nível socioeconômico, com benefícios para aquelas que frequentam instituições particulares^(9-11)^.

Para além da questão socioeconômica, outra questão importante de compreender é como se comporta o desenvolvimento da linguagem em indivíduos inseridos em diferentes culturas, visto que a diversidade linguística pode acompanhar a diversidade cultural^(12)^. Entretanto, estudos que busquem investigar essa relação são escassos, o que pode resultar em dificuldades no processo de avaliação linguística da criança^(13)^.

Os quilombolas são uma dentre as populações tradicionais do Brasil e consistem em um grupo étnico e minoritário dentro da população negra brasileira, frequentemente residindo em áreas rurais e distantes dos grandes centros urbanos. Em função das intensas desigualdades sociais a que está exposta e da localização geográfica das comunidades, essa população ainda sofre com a dificuldade de acesso a serviços de saúde e educação de qualidade^(14)^, o que pode interferir negativamente no desenvolvimento da linguagem e aumentar a vulnerabilidade social.

Buscar distinguir variações linguísticas culturais das alterações de linguagem que afetam diretamente o desenvolvimento da criança possibilita tanto a detecção de possíveis quadros de transtornos de linguagem, quanto ações e programas de intervenção na infância^(15)^.

Assim, considerando a ausência de estudos que enfoquem no desenvolvimento linguístico no contexto de comunidades quilombolas, este estudo tem por objetivo identificar o desempenho linguístico (vocabulário expressivo, fonologia e discurso) e cognitivo (memória de curto prazo verbal) de pré-escolares residentes de uma comunidade quilombola, levando em consideração sua idade e práticas familiares.

## MÉTODO

O estudo foi desenvolvido na comunidade quilombola de Capoeiras situada em Macaíba - RN, onde nos dias atuais residem cerca de 326 famílias, totalizando aproximadamente 1500 pessoas. A comunidade possui associação, cooperativa, igrejas católica e evangélica, duas casas de farinha, uma escola que atende o ensino infantil e fundamental I, posto de saúde e um ponto de cultura, que é um espaço onde as pessoas prestigiam as contribuições da cultura afro e rituais religiosos.

Os aspectos éticos do estudo foram aprovados pelo Comitê de Ética em Pesquisa do Hospital Universitário Onofre Lopes (CEP HUOL), da Universidade Federal do Rio Grande do Norte (UFRN), número CAAE 07867019.2.0000.5292. A participação das crianças foi autorizada pelos pais e/ou responsáveis mediante assinatura do Termo de Consentimento Livre e Esclarecido – TCLE.

### Participantes

A coleta de dados aconteceu entre maio e dezembro de 2019 e participaram desta pesquisa 24 pré-escolares com idade entre 4 anos (45,8%) e 5 anos (54,2%), cuja média foi de 58,6 (±6,4) meses. No grupo de 4 anos, 58% eram do gênero feminino, enquanto no grupo de 5 anos, 25%. Todos frequentavam a mesma instituição de ensino pública da comunidade e não havia queixa familiar com relação ao desenvolvimento de linguagem.

Com relação aos dados socioeconômicos, as famílias variaram entre as classes C2 e D-E, sendo majoritariamente D-E (86,4%). A escolaridade materna variou entre ensino fundamental I incompleto e ensino superior, com predomínio em fundamental I incompleto e fundamental II completo (30,4% cada); ao passo que a escolaridade paterna variou entre não alfabetizado e ensino médio completo, com predomínio do fundamental I completo (47,1%). Em todas as famílias a profissão mais frequente de mães e pais foi relacionada à agricultura (37,5%).

### Materiais e procedimentos

Inicialmente foi obtida autorização da prefeitura e dos líderes da comunidade quilombola. A seguir, as agentes comunitários de saúde convocaram as famílias para que reuniões fossem realizadas com os pais dos pré-escolares selecionados com o objetivo de esclarecer sobre os procedimentos do estudo e também assegurá-los sobre o anonimato e confidencialidade das informações obtidas.

Após obtido consentimento, os pais responderam a um curto questionário a respeito do desenvolvimento da criança e práticas no ambiente familiar, além de questões relacionadas ao poder de compra, acesso a bens e serviços e escolaridade, que permitem definir a classificação socioeconômica de acordo com Associação Brasileira de Empresas de Pesquisa^(16)^.

Para poder traçar o perfil de linguagem das crianças foi realizada uma bateria de avaliação que incluía a gravação em vídeo de tarefas de vocabulário expressivo, fonologia, narrativa e memória de curto prazo verbal. A coleta de dados ocorreu de forma individualizada e foi realizada na própria comunidade com duração média de 40 minutos, seguindo sempre a mesma ordem de aplicação.

Para avaliação do vocabulário foi utilizada a prova de Vocabulário Expressivo do ABFW^(17)^ e para responder aos objetivos deste estudo foram consideradas apenas as respostas classificadas como designação verbal usual (DVU), tanto em cada um dos campos semânticos, quanto no total na prova.

Para avaliação do sistema fonológico foi utilizada a prova de nomeação do teste de fonologia do ABFW^(17)^. Para caracterizar tal desempenho optamos por focar em processos fonológicos produtivos, assim foi considerado adequado quando não havia manutenção de nenhum processo fonológico produtivo além da idade de superação prevista, nem ocorrência daqueles considerados não esperados no desenvolvimento. Por oposição, o desempenho foi considerado alterado quando havia manutenção de algum processo fonológico produtivo além da idade de superação prevista e/ou ocorrência de processos considerados não esperados no desenvolvimento.

Para avaliação da memória de curto prazo verbal foi utilizado o teste de repetição de não palavras composto por 40 palavras inventadas divididas igualmente em mono, di, tri e polissilábicas^(18)^. Conforme proposto pelas autoras, para cada repetição correspondente à palavra-alvo ou que diferisse apenas por trocas fonológicas produtivas (previamente analisadas de acordo com a avaliação fonológica) foi atribuído 1 ponto. A seguir foram somados os acertos em cada parte do teste (monossílabos, dissílabos, trissílabos e polissílabos) e no total do teste (máximo 40). As porcentagens de acertos tanto parciais e a total foram calculadas e desempenho foi classificado como adequado ou abaixo do esperado de acordo com os valores propostos pelo teste.

Para eliciar o discurso foi utilizado o livro “*Frog, where are you*?”^(19)^, que é composto por 25 cenas em preto e branco, sem escrita e dispostas em sequência lógica. Foi solicitado que a criança explorasse as páginas e depois narrasse uma história com base no livro em mãos. As amostras de fala foram classificadas de acordo com o tipo de narrativa, a saber: descritivo, causal ou intencional^(20)^.

Para todos os testes foi realizada a transcrição do desempenho separadamente por dois pesquisadores distintos e quando encontrada divergência entre as análises, um terceiro pesquisador realizou conferência, garantindo assim precisão dos dados obtidos.

### Análise dos dados

Os dados obtidos foram tabulados e analisados no software estatístico SPSS versão 21. As variáveis categóricas (nível socioeconômico, escolaridade, práticas no ambiente familiar, desempenho em cada avaliação, tipo de discurso) foram descritas por meio da frequência de ocorrência e sua porcentagem. As variáveis numéricas (vocabulário, fonologia, memória de curto prazo verbal) foram submetidas à verificação da normalidade dos dados e, por não respeitarem a distribuição normal, foram descritas por meio da mediana e do intervalo interquartil.

Os dados do desempenho dos pré-escolares, faixas etárias e práticas familiares foram comparados por meio do teste exato de Fisher com correção de Bonferroni para as comparações múltiplas. Para estas análises foi necessário reclassificar os dados de discurso e práticas familiares, garantindo que teríamos apenas duas categorias a comparar. No caso do discurso foram agrupadas as classificações causal e intencional; e no caso das práticas familiares foram agrupadas as respostas “Às vezes e nunca” e “Frequentemente e sempre”. O nível de significância adotado foi de 5%.

## RESULTADOS

### Análise descritiva

Na Tabela 1 são apresentados os dados relacionados às práticas familiares, merecendo destaque para o fato que 52,6% dos responsáveis afirma ler às vezes para a criança e 70,0% afirma que a criança nunca lê livros sozinha. Na Tabela 2 são apresentados os dados relacionados aos itens que a criança possui e é possível verificar que nenhuma tem acesso ao computador em casa (Tabela 2).

**Tabela 1:** Distribuição de frequência da ocorrência das práticas familiares

	Nunca (%)	Às vezes (%)	Frequentemente (%)	Sempre (%)
Ajuda dever	11,1	44,4	5,6	38,9
Verifica dever	0,0	27,8	5,6	66,7
Lê para criança	10,5	52,6	10,5	26,3
Criança lê	70,0	15,0	5,0	10,0

**Tabela 2:** Distribuição de frequência dos itens que o pré-escolar tem acesso

Item	%
Instrumentos musicais	33,3
Jogos de tabuleiro	26,3
Dicionário	14,3
Livros	47,6
Computador	0,0
Jogos	47,6

Com relação ao desempenho geral nas diferentes áreas de linguagem, a maioria apresentou desempenho adequado no vocabulário (78,3%), na fonologia (79,2%), e discurso classificado como descritivo (63,6%), seguido pelo causal (31,8%). Com relação à memória de curto prazo verbal, a maioria apresentou desempenho abaixo do esperado para idade (82,6%).

### Comparação entre as idades e práticas familiares

Ao comparar a classificação do desempenho em cada área entre as faixas etárias apenas a fonologia e memória de curto prazo verbal diferiram, com o grupo de 5 anos tendo maior prejuízo (Tabela 3).

**Tabela 3:** Comparação da classificação do desempenho do pré-escolar em cada avaliação da linguagem

Variável	Classificação	4 anos (n,%)		5 anos (n,%)		p
Vocabulário	Adequado	8	72,7	11	84,6	0,415
	Abaixo do esperado	3	27,3	2	15,4	
Fonologia	Adequado	5	45,5	0	0,0	0,011*
	Alterado	6	54,5	13	100,0	
Memória de curto prazo verbal	Adequado	4	40,0	0	0,0	0,024*
	Abaixo do esperado	6	60,0	13	100,0	
Discurso	descritivo	7	70,0	7	58,3	0,454
	causal ou intencional	3	30,0	5	41,7	

* Diferença estatística (p=0,05) – teste exato de Fisher

Para comparar a classificação do desempenho em cada área entre as práticas familiares relacionadas ao dever escolar e à leitura utilizamos o teste exato de Fisher com uma correção de Bonferroni para dez efeitos planejados, resultando em um nível de significância de 0,005. Nenhuma destas comparações apresentou diferença estatística (Tabelas 4 e 5).

**Tabela 4:** Comparação da classificação do desempenho do pré-escolar em cada avaliação da linguagem de acordo com a classificação de práticas familiares relacionadas ao dever escolar.

Variável	Classificação	Ajuda dever				p	Verifica dever				p
		Às vezes ou nunca (n,%)		Frequentemente ou sempre (n,%)			Às vezes ou nunca (n,%)		Frequentemente ou sempre (n,%)		
Vocabulário	Adequado	8	80,0	7	87,5	0,588	5	100,0	10	76,9	0,350
	Abaixo do esperado	2	20,0	1	12,5		0	0,0	3	23,1	
Fonologia	Adequado	1	10,0	2	25	0,412	1	20,0	2	15,4	0,650
	Alterado	9	90,0	6	75		4	80,0	11	84,6	
Memória de curto prazo verbal	Adequado	3	30,0	1	12,5	0,382	3	60,0	1	7,7	0,044
	Abaixo do esperado	7	70,0	7	87,5		2	40,0	12	92,3	
Discurso	descritivo	5	54,6	5	71,4	0,451	2	40,0	8	72,7	0,242
	causal ou intencional	4	44,4	2	28,6		3	60,0	3	27,3	

* diferença estatística com correção de Bonferroni (p < 0,005) – teste exato de Fisher

**Tabela 5:** Comparação da classificação do desempenho do pré-escolar em cada avaliação da linguagem de acordo com a classificação de práticas familiares relacionadas à leitura.

Variável	Classificação	Lê para criança				p	Criança lê				p
		Às vezes ou nunca (n,%)		Frequentemente ou sempre (n,%)			Às vezes ou nunca (n,%)		Frequentemente ou sempre (n,%)		
Vocabulário	Adequado	10	83,3	6	84,2	0,704	12	70,6	3	100,0	0,399
	Abaixo do esperado	2	16,7	1	15,8		5	29,4	0	0,0	
Fonologia	Adequado	3	25,0	1	14,3	0,525	3	17,6	1	33,3	0,509
	Alterado	9	75,0	6	85,7		14	82,4	2	66,7	
Memória de curto prazo verbal	Adequado	4	33,3	0	0,0	0,128	4	23,5	0	0	0,491
	Abaixo do esperado	8	66,7	7	100,0		13	76,5	3	100	
Discurso	descritivo	7	63,6	4	66,7	0,661	9	56,3	3	100	0,227
	causal ou intencional	4	36,4	2	33,3		7	43,8	0	0	

* diferença estatística com correção de Bonferroni (p < 0,005) – teste exato de Fisher

## DISCUSSÃO

Este estudo buscou traçar o perfil de desempenho linguístico de pré-escolares quilombolas em provas de vocabulário expressivo, fonologia, discurso e memória de curto prazo verbal.

Inicialmente é importante ressaltar que estamos lidando com uma população considerada de baixa renda, majoritariamente da classe D-E, e com baixa escolaridade familiar, minoria de mães e pais que concluíram o ensino médio. Essa informação sozinha já nos alerta para possíveis consequências no desenvolvimento da linguagem, pois variáveis como vocabulário são apontadas como sensíveis ao ambiente^(6)^.

Ao considerarmos o desempenho na avaliação da linguagem, a maioria dos pré-escolares apresentou desempenho adequado para a faixa etária na prova de vocabulário expressivo, de acordo com os parâmetros de referência. Este resultado parece contrariar a literatura, entretanto ainda não há evidências de que o teste utilizado seja sensível e adequado à realidade local, o que poderia mascarar prejuízos mais leves. Vale ressaltar ainda que cerca de 20% dos pré-escolares apresentou desempenho abaixo do esperado e a comparação entre as idades, ainda que não tenha diferido, indicou menor porcentagem adequada no grupo de 4 anos. Embora a qualidade do ambiente escolar não tenha sido considerada, é possível que a inserção na escola tenha influenciado na ampliação de seu vocabulário^(6,21)^.

Na fonologia, mais de 75% pré-escolares apresentaram manutenção de processos fonológicos produtivos além da idade prevista para superação e foram considerados como tendo desempenho alterado. Ao comparar as idades houve diferença estatística, indicando que aos 5 anos todos apresentam desempenho alterado, ou seja, mantinham processos que já deveriam ter sido superados. Esses dados indicam que a maior parte das crianças desta amostra possui desempenho característico de um transtorno fonológico, ainda que não tenham sido observados processos fonológicos relacionados ao traço de sonoridade.

Mesmo não sendo uma medida puramente linguística, um dos objetivos do estudo foi investigar a memória de curto prazo verbal, pois ela mantém relação direta com a aquisição e a compreensão da linguagem e aprendizagem^(22,23)^. A maioria dos pré-escolares apresentou desempenho abaixo do esperado na tarefa de repetição de não palavras, sendo que nenhum sujeito com 5 anos foi classificado como adequado. Aqui é importante ressaltar que a tarefa utilizada propõe desconsiderar os processos fonológicos produtivos como erros^(18)^, o que implica que esse prejuízo não pode ser explicado pela fonologia, mas está relacionado ao armazenamento e recuperação da informação fonológica.

Esse achado precisa ser interpretado com cautela, pois se por um lado a avaliação se baseou apenas em um tarefa, por outro há vasta gama de literatura internacional apontando que prejuízos dessa natureza podem trazer impactos no percurso do desenvolvimento da linguagem e na vida escolar^(24)^ e são considerados inclusive como marca clínica do transtorno do desenvolvimento da linguagem^(25)^. Independente da natureza deste prejuízo e associado ao desempenho fonológico, este grupo demonstra risco para enfrentar dificuldades no processo de alfabetização e escolarização, pois habilidades de processamento fonológico têm um papel crucial para aprendizagem no início da educação formal^(26)^.

Com relação ao discurso a maioria dos pré-escolares produziu um discurso descritivo, padrão que se manteve ao considerar as faixas etárias. Apesar de não dispormos de dados de referência brasileiros, o discurso descritivo é simples e não demonstra refinamento das habilidades narrativas^(20)^, que já poderiam estar presentes na faixa etária estudada.

Aqui é interessante pontuar um aspecto qualitativo, pois para eliciar o discurso foi utilizado um livro com figuras e nos chamou atenção a falta de familiaridade dos participantes com o manuseio de um livro. Quando entrevistadas sobre práticas familiares, 52,2% das mães afirmou que “às vezes” lê para a criança e 46,7% afirmou que a criança tem livros, ao passo que 70,0% afirma que a criança nunca lê livros sozinha. É importante ressaltar que não se espera uma leitura efetiva nessas idades, mas que a criança conseguisse manipular o livro adequadamente. Ainda que seja uma informação baseada em relato, a falta de familiaridade com livros nos parece estar associada à baixa escolaridade dos pais e, por consequência, ao valor que é atribuído a tais práticas nesta comunidade.

A ausência de diferenças estatísticas entre a frequência de práticas familiares positivas e o desempenho linguístico nesta amostra nos parece estar relacionada tanto ao número restrito de sujeitos e à necessidade de reagrupar os dados, quanto ao fato das famílias apresentarem certa homogeneidade no que refere às variáveis socioeconômicas. A quantidade e a qualidade dos estímulos que a criança recebe dependem, porém, de suas condições de vida e das características da comunidade em que vive^(27)^. Considerando que é possível observar diferenças no processo de aprendizagem em crianças que crescem em famílias que incentivam o contato com a linguagem escrita^(28)^, este é outro achado que reforça a vulnerabilidade relacionada ao desenvolvimento de habilidades comunicativas dos pré-escolares desta comunidade.

De forma geral, estes achados apontam para a importância de investimentos tanto no monitoramento do desenvolvimento comunicativo na primeira infância, quanto na implementação de programas de intervenção na idade ideal que possam minimizar o impacto que um ambiente menos favorável no desenvolvimento das crianças nesta comunidade. Programas baseados em resposta à intervenção em parceria com a educação, por exemplo, parecem uma alternativa de baixo custo que poderia trazer benefícios à comunidade^(29)^.

Uma análise que não foi possível neste momento, mas nos parece promissora, é a comparação com o desempenho de um grupo residente na capital do estado onde o estudo foi realizado e que apresente maior variabilidade socioeconômica. Esta análise nos auxiliará a compreender se os instrumentos utilizados podem ter interferido demasiadamente nestes resultados, afinal é importante considerar a efetividade de testes de desempenho linguístico para minorias populacionais devido a grande variabilidade regional e cultural^(30)^.

Por ser um estudo transversal realizado apenas com uma avaliação de cada criança, não foi possível aprofundar a investigação sobre a influência cultural e o desenvolvimento da linguagem. Entretanto, os resultados aqui apresentados possibilitam indicar que os pré-escolares que vivem nesta comunidade quilombola apresentam desenvolvimento linguístico que os permite ter uma comunicação funcional, apesar de haver indicativos de risco para dificuldades no processo de inserção escolar. Assim, considerando que se trata de um contexto socioeconômico vulnerável, sinalizamos a importância do investimento em programas de acompanhamento e estimulação, assim como contar com o apoio da escola.

Por fim, esse estudo traz contribuição potencial para a prática clínica em regiões com menor investimento governamental e ressalta a importância de pensarmos políticas públicas para a primeira infância que visem o desenvolvimento do pleno potencial de crianças inseridas em populações com risco para alterações cognitivas e de transtornos de linguagem.

## CONCLUSÃO

Os pré-escolares deste estudo apresentaram desempenho compatível com os valores de referência no vocabulário, porém seu sistema fonológico demonstra alteração devido à manutenção de processos fonológicos produtivos. Suas habilidades discursivas são descritivas e seu desempenho na memória de curto prazo fonológica está prejudicado. Ainda que estas crianças apresentem uma comunicação funcional, elas apresentam um perfil de desenvolvimento da linguagem e de habilidades cognitivas mais vulnerável e que pode repercutir em sua trajetória escolar.
